# Factors associated with infection and hospitalization due to COVID-19 in Nursing professionals: a cross-sectional study

**DOI:** 10.1590/1518-8345.5593.3524

**Published:** 2022-05-16

**Authors:** Vilanice Alves de Araújo Püschel, Jack Roberto Silva Fhon, Lilia de Souza Nogueira, Vanessa de Brito Poveda, Larissa Bertacchini de Oliveira, Marina de Góes Salvetti, Cassiane de Santana Lemos, Camila Quartim de Moraes Bruna, Fernanda Rodrigues Lima, Ana Beatriz Pandolfo da Silva, Fábio da Costa Carbogim

**Affiliations:** 1 Universidade de São Paulo, Escola de Enfermagem, São Paulo, SP, Brasil.; 2 Universidade de São Paulo, Faculdade de Medicina, Hospital das Clínicas, Instituto do Coração, São Paulo, SP, Brasil.; 3 Bolsista da Coordenação de Aperfeiçoamento de Pessoal de Nível Superior (CAPES), Brasil.; 4 Hospital Municipal Infantil Menino-Jesus, São Paulo, SP, Brasil.; 5 Universidade Federal de Juiz de Fora, Escola de Enfermagem, Juiz de Fora, MG, Brasil.

**Keywords:** Nursing, Coronavirus Infections, Nurse Practitioners, Hospitalization, Occupational Health, Surveillance of the Workers Health, Enfermagem, Infecções por Coronavírus, Profissionais de Enfermagem, Hospitalização, Saúde do Trabalhador, Vigilância em Saúde do Trabalhador, Enfermería, Infecciones por Coronavirus, Profesionales de Enfermería, Salud Laboral, Vigilancia de la Salud del Trabajador

## Abstract

**Objective:**

to identify factors associated with infection and hospitalization due to COVID-19 in nursing professionals.

**Method:**

a cross-sectional study carried out with 415 nursing professionals in a hospital specialized in cardiology. The sociodemographic variables, comorbidities, working conditions and issues related to illness due to COVID-19 were evaluated. Chi-Square, Fisher’s, Wilcoxon, Mann-Whitney and Brunner Munzel tests were used in data analysis, as well as Odds Ratio for hospitalization, in addition to binary logistic regression.

**Results:**

the rate of nursing professionals affected by COVID-19 was 44.3% and the factors associated with infection were the number of people living in the same household infected by COVID-19 (OR 36.18; p<0.001) and use of public transportation (OR 2.70; p=0.044). Having severe symptoms (OR 29.75), belonging to the risk group (OR 3.00), having tachypnea (OR 6.48), shortness of breath (OR 5.83), tiredness (OR 4.64), fever (OR 4.41) and/or myalgia (OR 3.00) increased the chances of hospitalization in professionals with COVID-19.

**Conclusion:**

living in the same household as other people with the disease and using public transportation increased the risk of infection by the new coronavirus. The factors associated with the hospitalization of contaminated professionals were presence of risk factors for the disease, severity and type of the symptoms presented.

## Resumo

**Objetivo:**

identificar fatores associados à contaminação e internação hospitalar por COVID-19 em profissionais de enfermagem.

**Método:**

estudo transversal, realizado em hospital especializado em cardiologia, com 415 profissionais de enfermagem. Foram avaliadas as variáveis sociodemográficas, comorbidades, condições de trabalho e questões relacionadas ao adoecimento pela COVID-19. Na análise dos dados, utilizaram-se os testes Qui-Quadrado, Fisher, Wilcoxon, Mann-Whitney e Brunner Munzel, a razão de chances para internação hospitalar, além de regressão logística binária.

**Resultados:**

a taxa de profissionais de enfermagem acometidos pela COVID-19 foi de 44,3% e os fatores associados à contaminação foram o número de pessoas no mesmo domicílio com COVID-19 (OR 36,18; p<0,001) e o uso de transporte público (OR 2,70; p=0,044). Ter sintomas graves (OR 29,75), pertencer ao grupo de risco (OR 3,00), apresentar taquipneia (OR 6,48), falta de ar (OR 5,83), cansaço (OR 4,64), febre (OR 4,41) e/ou mialgia (OR 3,00) aumentou as chances de internação hospitalar dos profissionais com COVID-19.

**Conclusão:**

habitar o mesmo domicílio que outras pessoas com a doença e usar transporte público aumentou o risco de contaminação pelo novo coronavírus. Os fatores associados à internação hospitalar dos profissionais contaminados foram a presença de fatores de risco para doença, a gravidade e o tipo dos sintomas apresentados.

## Resumen

**Objetivo:**

identificar los factores asociados al contagio y la hospitalización por COVID-19 en los profesionales de enfermería.

**Método:**

estudio transversal, realizado en un hospital especializado en cardiología, con 415 profesionales de enfermería. Se evaluaron las variables sociodemográficas, comorbilidades, condiciones de trabajo y preguntas relacionadas con la enfermedad por COVID-19. En el análisis de los datos, se utilizaron las pruebas de Chi-Cuadrado, Fisher, Wilcoxon, Mann-Whitney y Brunner Munzel, la razón de chance para la hospitalización, además de la regresión logística binaria.

**Resultados:**

la tasa de profesionales de enfermería afectados por el COVID-19 fue del 44,3% y los factores asociados al contagio fueron el número de personas en una misma vivienda con COVID-19 (OR 36,18; p<0,001) y el uso de transporte público (OR 2,70; p=0,044). Presentar síntomas graves (OR 29,75), pertenecer al grupo de riesgo (OR 3,00), tener taquipnea (OR 6,48), dificultad para respirar (OR 5,83), cansancio (OR 4,64), fiebre (OR 4,41) y/o mialgia (OR 3,00) aumentó las chances de hospitalización de los profesionales con COVID-19.

**Conclusión:**

vivir en el mismo domicilio que otras personas que tienen la enfermedad y utilizar el transporte público aumentó el riesgo de contagio por el nuevo coronavirus. Los factores asociados a la hospitalización de los profesionales contagiados fueron la presencia de factores de riesgo para enfermarse, la gravedad y el tipo de síntomas presentados.

## Introduction

Acute respiratory syndrome 2019 (COVID-19) is caused by a new coronavirus, SARS-CoV-2, from the family of coronaviruses (CoV), responsible for infectious manifestations ranging from a common cold to Severe Acute Respiratory Syndrome (SARS)^1^ ^-^ ^2^. The disease had its first cases identified in China at the end of 2019 and, in a few months, spread across the world^1^. 

A research study pointed out that, at the beginning of the pandemic, nearly 14% of the infected cases were serious and required hospitalization; in addition to that, 1.7% underwent treatment by invasive mechanical ventilation and 2.6% died^3^. 

Data from the World Health Organization (WHO) taken on November 13^th^, 2021, indicate 252,728,611 confirmed COVID-19 cases worldwide, with the highest numbers in the United States (47,013,894 cases), followed by India (34,426,036 cases) and Brazil (21,939,196 cases). In relation to mortality, from a global point of view, COVID-19 has already caused 5,092,908 deaths, with 762,614 in the United States, 610,491 in Brazil and 463,245 in India^4^.

Prevention of transmission during care and treatment of the patients depends on the effective use of Personal Protective Equipment (PPE) items, which must include a mask, goggles or face shield, gloves and apron, exclusive to the care environment^5^ ^-^ ^6^. The complexity of donning, associated with the fear of infection and, often, PPE scarcity, increases tension and stress in healthcare professionals who work against coronavirus^5^ ^-^ ^6^. 

In 2020, the scarcity of testing resources, uncertainty about the prognostic factors, the unavailability of vaccines, the imposition of unknown public health measures, significant financial losses and conflicting messages from the authorities were reasons for anguish and stress in the healthcare professionals^6^.

Nurses and midwives represent nearly 50% of the healthcare workforce. Of the 43.5 million healthcare workers in the world, an estimated 20.7 million are nurses and midwives^7^. In Brazil, data obtained from the Federal Nursing Council website indicate that there are 2,305,946 registered and active nursing professionals, of which 565,458 are nurses, 1,320,239 nursing technicians, 419,959 nursing assistants and 290 midwives^8^.

Thus, nursing is at the forefront of the care provided to patients with COVID-19 and plays a central role in clinical care, education, prevention and control of the disease^9^, facing the fear of contagion, dying or infecting their family members.

A research study that analyzed cases and deaths due to COVID-19 in nursing professionals in Brazil showed a higher number of cases in the Southeast region, with higher lethality for the age group between 41 and 50 years old and males^10^.

A cross-sectional study, conducted with Iranian healthcare professionals, described the highest rate of COVID-19 infection among nurses (51.3%). Nearly one third of the professionals were asymptomatic and, for the symptomatic ones, the most frequent clinical features were myalgia (46%) and cough (45.5%)^11^.

Due to the sudden outbreak of the disease, nurses had only brief training to care for COVID-19 patients and many professionals were distanced from work due to flu-like symptoms and suspected or even confirmed infection^9^ ^,^ ^11^. The absence of healthcare professionals generates an overload in the services and this factor, added to PPE shortage, raises healthcare professionals’ tension^6^ ^,^ ^12^.

In the crisis context caused by the new coronavirus, the working conditions and the illness of nursing professionals, knowing the factors associated with infection, illness by COVID-19 and the need for hospitalization of nursing professionals can contribute to the adoption of protective measures for healthcare professionals both in this and in possible future health crises. In this way, healthcare service managers can gain subsidies to support occupational health actions, such as control of the professionals’ comorbidities; adequate staffing to manage the exposure of the most vulnerable professionals and management of the work overload of the work teams, as well as implementation of continuing education actions that guide proper PPE use by the professionals.

This study hypothesized that lack of PPE and presence of comorbidities among nursing professionals can be associated with COVID-19 infection. Thus, the research aimed to identify factors associated with infection and hospitalization due to COVID-19 in nursing professionals. 

## Method

### Type of study

This is an analytical, cross-sectional and observational study based on the Strengthening the Reporting of Observational Studies in Epidemiology (STROBE) guidelines^13^. Cross-sectional studies are characterized as those that evaluate the outcome and exposure of the participants at the same time, with selection of individuals based only on the study inclusion and exclusion criteria^14^.

### Study locus, population and sample

The study was carried out in a teaching hospital specialized in cardiopneumology in the city of São Paulo-SP, Brazil. The institution is a reference center for the care of patients with complex cardiological and pulmonological conditions and, since June 2020, it has become a reference for the care of patients with COVID-19 in the city of São Paulo, reason why it was chosen for development of the research. 

The hospital consists of 535 beds distributed in seven inpatient units and 157 beds in a high-complexity Intensive Care Unit (ICU), in addition to having 14 operating rooms, seven hemodynamic and electrophysiological studies rooms, 12 high-complexity diagnostic rooms and 60 medical offices. For the exclusive care of patients with COVID-19, 50 ICU beds and 60 inpatient unit beds were made available.

The hospital has 1,283 nursing professionals, of which 125 are assistants, 718 are nursing technicians and 440 are nurses. During the data collection period, 248 professionals were on vacation or medical/maternity leave. The sample was for convenience and consisted of nurses and nursing technicians/assistants.

### Inclusion and exclusion criteria

Nursing professionals who have worked at the institution for at least one month in the Intensive Care Units, Inpatient Units, Surgical Center, Hemodynamics, Emergency Room, Diagnostic Imaging Service and Outpatient Clinic were included. Professionals who were on vacation or on sick leave (not related to COVID-19) during the data collection period were excluded. 

### Study variables

The dependent variables were as follows: infection by COVID-19 and need for the professionals to be hospitalized due to COVID-19. The independent variables included sociodemographic data (gender, age, race, marital status, religion, place of residence, number of people in the household), training (schooling and courses in the field of nursing), work-related (professional category, function, income, means of transport to work, sector in which they operate, working time in the institution, working hours, specific training for the care of patients with COVID-19, availability of PPE, whether they had any other assistance employment contract, leave of absence for work-related emotional reasons and provision of institutional mental health support), health conditions (comorbidities and/or whether thy presented risk group factors defined by the WHO - aged people over 60 years old, smokers, people with cardiovascular, respiratory, renal or cancer diseases, diabetics, immunosuppressed people, pregnant women and obese individuals with a Body Mass Index greater than 40). In addition, for those who contracted COVID-19, diverse information about the severity and type of the symptoms presented and the need for intensive care was included. 

### Data collection instrument

For data collection, a checklist-type instrument was developed, consisting of two parts. The first included sociodemographic variables, aspects related to housing, comorbidities, working conditions and information about the institution’s work. The second part consisted of questions related to infection by COVID-19 and the need for the professionals to be hospitalized due to COVID-19. The instrument was built using as a reference guidelines that instruct about the good practices related to observational studies^13^, risk factors^15^ and the professionals’ biosafety^16^ ^-^ ^17^, as there were still no validated instruments that could be used. 

### Data collection and period

The data collection instrument was prepared in a survey format in Research Electronic Data Capture (RedCap), a system that guarantees the security of the information recorded and it was forwarded to all nursing professionals via messaging apps, in addition to providing the link to the instrument on the computers of all units where the data were collected. 

The researchers were present in all units and periods, providing the link and guiding the professionals. In addition to that, a QR Code was created to facilitate the professionals’ access to the instrument.

The data were collected in November and December 2020.

### Data treatment and analysis 

The data were analyzed in the R statistical program, version 4.1.1, with the support of a professional statistician. When comparing the groups (whether or not there was infection by the new coronavirus and whether or not there was a need for hospitalization), for the nominal variables of the study, Pearson’s Chi-Square or Fisher’s Exact tests were used (in the cases where the expected frequency in at least one of the boxes in the contingency table was less than 5). As for the discrete and continuous quantitative variables, the groups were compared using the Wilcoxon, Mann-Whitney and Brunner Munzel tests. To identify the factors associated with illness due to COVID-19, binary multiple logistic regression was applied and all the independent variables described above were simultaneously inserted into the model, whose predictive capacity was evaluated by the area under the Receiver Operator Characteristic curve (AUC-ROC). The Variance Inflation Factor (VIF) was applied to identify the presence of multicollinearity in the variables of this model and a VIF value below 5 was interpreted as absence of collinearity. For hospital admission, the Odds Ratio was calculated for the variables that were significant in the bivariate analyses. The significance level adopted was 5%. 

### Ethical aspects

The research was approved by the institution’s Ethics Committee (opinion No. 4,072,114) and all participants signed the Free and Informed Consent Form (FICF). 

## Results

A total of 415 nursing professionals participated in the study (86.7% female; mean age of 36.7 years old), with a higher frequency of professionals of white (47.8%) and mixed race (33.2%) and mid-level training, that is, nursing technicians and assistants (53.7%). The participants lived in households with an approximate mean of three people, most lived in the capital of São Paulo (71.8%) and used public transportation (78.3%) when commuting to work (Table 1). In relation to Table 1, as some items were not answered by all study participants, the “n” of the variable that did not result in 415 is explained after the description of the variable itself.

**Table 1:** Distribution of the nursing professionals according to demographics, schooling, housing and transportation. São Paulo, SP, Brazil, 2020

Variable	n (%)	Mean (SD)^*^
**Gender (n=407)**		
Female	353 (86.7)	
Male	54 (13.3)	
Age^†^		36.7 (10.0)
**Race (n=404)**		
White	193 (47.8)	
Brown	134 (33.2)	
Black	57 (14.1)	
Asian	18 (4.4)	
Indigenous	2 (0.50)	
**Training level (n=410)**		
Average	220 (53.7)	
Higher education	190 (46.3)	
Number of people in the household^†|^		3.1 (1.41)
**Area of residence^†^**		
São Paulo capital	298 (71.8)	
Greater São Paulo^‡^	91 (22)	
ABC^§^	23 (5.5)	
Inland/Coast	3 (0.7)	
**Transportation means^†||^**		
Public	325 (78.3)	
Private car	136 (32.7)	
By app	21 (5.1)	
Walking	12 (2.9)	
Others	6 (1.4)	

*SD = Standard Deviation; ^†^n = 415; ^‡^Greater São Paulo = It consists of 35 municipalities, excluding *ABC Paulista* (analyzed separately); ^§^ABC = Santo André, São Bernardo do Campo, São Caetano do Sul and Diadema; ^||^It allowed more than one answer

Among the study participants, 110 (26.5%) were classified as a risk group for COVID-19. In addition to that, the main comorbidities identified were respiratory diseases (5.5%), cardiovascular diseases (5.1%), obesity (4.8%) and diabetes (3.1%). As for the respiratory and cardiovascular diseases, asthma (n=15) and systemic arterial hypertension (n=21) stood out (Table 2).

**Table 2:** Distribution of the nursing professionals (n=110) according to risk conditions for COVID-19. São Paulo, SP, Brazil, 2020

Risk conditions for COVID-19*	n	%
Cardiovascular disease	21	5.1
Systemic arterial hypertension	21	5.1
Heart failure	2	0.5
Congenital cardiopathy	2	0.5
Arrhythmia	1	0.2
Coronary syndrome	1	0.2
Respiratory disease	23	5.54
Asthma	15	3.6
Bronchitis	7	1.7
COPD^†^	1	0.2
Diabetes	13	3.1
Neoplasm	3	0.7
Immunosuppression due to medication	1	0.2
Autoimmune disease	6	1.4
Pregnancy	7	1.7
Age over 60 years old	14	3.4
Smoker	10	2.4
Obesity	20	4.8
Others	13	3.1

*It allowed more than one answer; ^†^COPD = Chronic Obstructive Pulmonary Disease

Twenty-three professionals (5.5%) worked in more than one institution and the mean period of time they have worked at the study hospital was 7.5 years (SD=8.6). A total of 15 sectors of activity were identified, with emphasis on the adult inpatient unit (23.5%), surgical ICU (17.1%) and emergency room (12.0%). 

Most of the participants (52.8%) provided exclusive care to patients with COVID-19 and approximately 78% of the institution’s professionals received training to serve this type of clients. More than half of the professionals reported lack of some type of PPE at the institution (50.1%), especially N95/PFF2 (37.1%) or surgical (29.9%) masks, waterproof aprons (19.0%) and face shields/goggles (2.4%).

A total of 184 (44.3%) nursing professionals were infected with COVID-19 and infection was associated with the number of people with the disease living in the same household (p<0.001), use of public transportation (p=0.04), work in another institution (p=0.012), sector of work (p<0.001), lack of PPE (p=0.033) and lack of N95/PFF2 mask (p=0.029). 

Among the variables that were associated with infection by COVID-19, living in the same household with other people with the disease increased 36.18 times the chance of contracting COVID-19 and use of public transportation increased 2.70 times the risk of infection, when compared to those who did not need this type of transportation (Table 3).

**Table 3:** Factors associated with infection by the new coronavirus in nursing professionals (n=415). São Paulo, SP, Brazil, 2020

	CI* for OR 95%					
	OR^†^	SE	LL^‡^	UL^§^	p-value	VIF^||^
Male Gender	1.87	1.66	0.69	5.08	0.216	1.149
Age	0.98	1.03	0.93	1.03	0.400	2.439
White race	0.70	2.48	0.13	4.50	0.694	1.639
Indigenous race	0.21	83.25	0.00	72.10	0.721	
Brown race	0.57	2.61	0.09	4.05	0.557	
Black race	0.63	2.72	0.10	4.82	0.645	
Area of residence - Inland of São Paulo	0.58	1.48	0.27	1.25	0.174	1.192
Number of people per household	0.89	1.13	0.70	1.13	0.335	1.214
Number of people with COVID-19 in the household	36.18	1.43	18.70	76.38	< 0.001	1.261
Working time in the hospital	1.00	1.00	0.99	1.00	0.901	2.189
Exclusive care for patients with COVID-19	1.06	1.43	0.53	2.15	0.860	1.172
Mid-level professional	0.97	1.48	0.45	2.09	0.937	1.404
Uses public transportation	2.70	1.64	1.05	7.28	0.044	1.403
Uses private car	1.78	1.51	0.79	4.05	0.166	1.402
Uses transportation by app	1.11	2.09	0.26	4.67	0.887	1.193
Walks to work	1.84	2.57	0.28	11.34	0.518	1.151
Uses a different kind of transportation means	3.80	3.00	0.34	29.33	0.224	1.126
Received training to respond to COVID-19	1.08	1.50	0.49	2.43	0.842	1.151
Has another job	2.27	2.03	0.58	9.51	0.247	1.131
Surgical mask not available	1.01	1.62	0.39	2.63	0.976	1.944
N95/PFF2 mask not available	1.51	1.55	0.64	3.62	0.348	1.747
Face shield/goggle not available	1.24	1.79	0.40	3.88	0.711	1.385
Hat not available	0.69	1.96	0.19	2.64	0.580	1.440
Waterproof apron not available	0.77	1.70	0.27	2.18	0.630	1.712
Gloves not available	2.54	2.18	0.55	11.65	0.230	1.292
Belongs to the risk group for COVID-19	1.50	1.72	0.52	4.38	0.457	2.184
Diabetes	0.84	3.51	0.07	9.18	0.889	1.255
Neoplasm	2.58	8.06	0.04	197.70	0.650	1.236
Uses immunosuppressant	368,480	Not estimable			0.988	1.000
Has an autoimmune disease	0.73	5.20	0.02	12.67	0.848	1.125
Pregnancy	0.16	4.55	0.01	3.08	0.235	1.225
Age over 60	0.73	3.32	0.07	7.65	0.789	1.584
Smoker	0.36	3.01	0.03	2.73	0.350	1.278
Obesity	0.92	2.47	0.16	5.45	0.930	1.355
Other risks	0.36	2.79	0.04	2.56	0.321	1.320

*CI = Confidence Interval; ^†^OR = Odds Ratio; ^‡^LL = Lower Limit; ^§^UL = Upper Limit; ^||^VIF = Variance Inflation Factor

The VIF values indicated absence of collinearity between the variables of the model related to the factors associated with infection by COVID-19 in the professionals (Table 3), which presented excellent predictive capacity according to the AUC-ROC result: 0.958. 

Table 4 presents the main signs and symptoms presented by the professionals who had COVID-19, as well as the need for hospitalization and ICU admission. It is observed that the majority presented mild symptoms (68.7%), especially headache (63.5%), tiredness (62.5%), anosmia (58.6%) and ageusia (55.9%). Of the 184 professionals who contracted the disease, 16 (8.7%) required hospitalization for treatment and four (2.2%) needed intensive care.

**Table 4:** Distribution of the nursing professionals (n=184) according to COVID-19 severity and main symptoms, need for hospitalization and ICU* admission. São Paulo, SP, Brazil, 2020

Variables	n (%)
**Severity of the symptoms (n=179)**	
Asymptomatic	32 (17.9)
Mild	123 (68.7)
Severe	24 (13.4)
**Main symptoms^†^**	
Headache	117 (79.6)
Tiredness	115 (78.2)
Anosmia	108 (73.5)
Ageusia	103 (70.1)
Coughing	84 (57.1)
Myalgia	82 (55.8)
Fever	80 (54.4)
Fatigue	78 (53.1)
**Hospitalization**	
Yes	16 (8.7)
No	168 (91.3)
**ICU admission**	
Yes	4 (2.2)
No	180 (97.8)

*Intensive Care Unit ^†^It allowed more than one answer

When comparing the 184 professionals with COVID-19, according to whether or not they needed to be hospitalized, there was a significant difference between the groups in terms of belonging to the risk group for COVID-19 (p=0.032) and having severe symptoms of the disease (p<0.001), in addition to the presence of fever (p=0.008), shortness of breath (p<0.001), tiredness (p=0.031), tachypnea (p<0.001) and/or myalgia (p=0.042). 

These variables, which showed an association with hospitalization, were individually tested as to the Odds Ratio for hospitalization. It was verified that the presence of severe symptoms, tachypnea or shortness of breath increased the chance of hospitalization by 29.75, 6.48 and 5.83 times, respectively. Tiredness, fever, myalgia and belonging to the risk group also contributed to hospitalization, with an Odds Ratio of less than 5 (Table 5).

**Table 5:** Odds Ratio for the hospitalization of nursing professionals (n=415), based on the variables that showed an association in the univariate analysis. São Paulo, SP, Brazil, 2020

Variables	OR^†^	95% CI^*^		p-value
		Lower limit	Upper limit	
Belonging to the risk group	3.00	1.06	8.49	0.032^‡^
Severe symptoms	29.75	8.26	106.77	<0.001^§^
Fever	4.41	1.36	14.25	0.008^‡^
Shortness of breath	5.83	1.92	17.70	<0.001^‡^
Tiredness	4.64	1.02	21.09	0.031^‡^
Tachypnea	6.48	2.15	19.51	<0.001^‡^
Myalgia	3.00	1.00	9.03	0.042^‡^

^*^CI = Confidence interval; ^†^OR = Odds Ratio; ^‡^Pearson’s Chi-Square Test; ^§^Fisher’s Exact Test

## Discussion

Throughout the COVID-19 pandemic, hundreds of professionals were contaminated and many died as a result of the disease. Although it is not always possible to establish the care provided as the source of infection, even when the professionals directly care for patients infected with SARS-CoV-2, a number of research studies indicate a higher risk for healthcare workers when compared to the general population^11^ ^-^ ^18^. 

It is estimated that before mass vaccination, nearly 14% of the world’s cases were in healthcare professionals, from different areas of activity^19^. However, even with the emergence of the SARS-CoV-2 variants, worldwide vaccination was essential to control the COVID-19 pandemic in the general population and among professionals. In this sense, a cohort consisting of 194,362 family members of healthcare professionals and 144,525 healthcare workers showed that the risk of infection by COVID-19 was lower after the second dose for family members [HR - Hazard Ratio - 0.46 (95% CI 0.30-0.70)] and for healthcare professionals [HR 0.08 (95% CI 0.04-0.17)]^20^.

During the most critical phase of the pandemic or pre-vaccination period, there was a reduction in the healthcare workforce due to infection and illness, which exerted a significant social and economic impact on the health system^19^ ^,^ ^21^. In this context, this research explored factors associated with the illness of nursing professionals due to COVID-19 in the period before vaccination in the country. 

The current study allowed identifying that the use of public transportation increased the chances of the professionals being contaminated by SARS-CoV-2, similarly to other studies that investigated the relationship between public transportation and the risk of infection by COVID-19^22^ ^-^ ^23^. 

A Chinese study that assessed the transmission risk of the new coronavirus in train passengers concluded that contagion on these trips is high, although the risk is influenced by the passenger’s exposure time and location and can be minimized by increasing the distance between seats, reducing passenger density and applying personal hygiene measures^23^.

An integrative literature review analyzed the risks of occupational illness in healthcare professionals who cared for patients with COVID-19. The authors analyzed 19 studies and highlighted the importance of proper PPE use, hand hygiene and the environment, suggesting that the healthcare services should plan the turnover of the professionals in the care of infected or suspected patients, reducing the time of exposure to the virus whenever possible^24^. 

Although broad public policies are difficult to implement, some initiatives can be considered with the purpose of mitigating infection and transmission, such as the use of masks, distancing and adequate ventilation^22^. Other possibilities would be granting of free transportation by the private sector and implementation of exclusive transportation means for healthcare professionals^25^ ^-^ ^26^.

Another variable that significantly increased the chance of contracting the disease was living in the same household as other people diagnosed with COVID-19. It is noteworthy that, in this study, as in others, it was not possible to identify whether infection went from the professionals to the residents of the same environment or vice versa. 

Although rapid identification of cases through surveillance and diagnostic testing makes rapid isolation possible, isolation often needs to be done at home with family members and other close people. Providing accommodations for the quarantine of infected front-line professionals is an example of a measure that can help reduce possible infection in the community^27^.

The need for the contaminated professionals to be hospitalized was associated with belonging to the risk group and to the presence of severe symptoms of the disease, factors that were also observed in other studies that indicated the presence of comorbidities and respiratory symptoms as predictors for the hospitalization of patients affected by COVID-19^28^ ^-^ ^31^.

Special attention, with relocation or even distancing of professionals at greater risk, such as elderly individuals or those with comorbidities, has been used as a strategy in an attempt to prevent worsening of the pandemic. Thus, collecting and managing the teams’ healthcare data is an important measure to be considered. 

In this study, the low incidence of professionals who needed to be admitted to the ICU (2.2%) corroborates already known data that report rates between 10% and 20% requiring intensive care, with only 3% to 10% of them requiring intubation^32^.

A cohort study developed in Spain compared the outcomes of healthcare workers and of the general population hospitalized due to COVID-19. The results showed that comorbidities and severe radiological findings were more frequent in the general population, and no significant difference was found between the need for ventilatory support and ICU admission between the two groups. However, the incidence of sepsis and mortality was significantly higher in the general population than among the healthcare professionals^33^.

Given the characteristic of the current study, in which the professionals themselves answered about their health conditions, death was not a variable under evaluation, although it is known that there is high mortality for patients who need ICU admission^34^ ^-^ ^35^. 

In the current study, a relationship was established between infection and lack of PPE. It is believed that the occupational risk imposed by the lack of such equipment should be avoided, and the availability of adequate PPE should receive special attention in local management of the pandemic^36^.

The current study has strengths and limitations that must be pointed out. The main advance in knowledge was the identification of factors associated with infection and illness by COVID-19, in addition to the variables associated with the hospitalization of nursing professionals. The findings have potential to be used as a reference in the assessment and comparison of health risk factors among front-line nursing professionals, both in the current context and in future pandemic contexts. The results may contribute to future studies analyzing factors associated with infection, illness and hospitalization of healthcare workers due to COVID-19. 

Among the limitations, the sample size established for convenience and in a non-probabilistic way stands out. In addition to that, data collection took place in only one institution, through self-report, which may incur in some degree of subjective bias.

## Conclusion

The study made it possible to identify factors associated with the infection of nursing professionals by COVID-19. Living in the same household as other people with the disease and using public transportation increased the professionals’ risk of infection. In addition to that, lack of PPE was related to infection of the nursing team, identifying the need to manage material resources in the healthcare services to guarantee the supply of adequate human resources during the pandemic.

The need for hospital admission among professionals who got infected with COVID-19 was low and was associated with belonging to the risk group, having severe symptoms of the disease and having fever, shortness of breath, fatigue, tachypnea and/or myalgia. Thus, the presence of comorbidities stands out as a significant factor for the infection of nursing professionals and reflects the need for occupational health actions that assist in the management of these health problems.

It is recommended to conduct new studies that comparatively analyze healthcare institutions and systematic reviews that synthesize the factors associated with infection of nursing professionals by COVID-19 and the measures adopted.

## References

[1] World Health Organization (2020). Coronavirus disease (COVID-19) pandemic..

[2] Yin Y, Wunderink RG (2018). MERS, SARS and other coronaviruses as causes of pneumonia. Respirol.

[3] Telle KE, Grøsland M, Helgeland J, Håberg SE (2021). Factors associated with hospitalization, invasive mechanical ventilation treatment and death among all confirmed COVID-19 cases in Norway: Prospective cohort study. Scand J Public Health.

[4] Johns Hopkins University and Medical (2020). COVID-19 Dashboard.

[5] Cambien G, Guihenneuc J, Fouassin X, Castel O, Bousseau A, Ayraud-Thevenot S (2021). Management of donations of personal protective equipment in response to the massive shortage during the COVID-19 health crisis: providing quality equipment to health care workers. Antimicrob Resist Infect Control.

[6] Haegdorens F, Franck E, Smith P, Bruyneel A, Monsieurs KG, Van Bogaert P (2021). Sufficient personal protective equipment training can reduce COVID-19 related symptoms in healthcare workers: a prospective cohort study. Int J Nurs Stud.

[7] World Health Organization (2016). Global strategic directions for strengthering nursing and midwifery 2016-2020..

[8] Federal Nursing Council (2020). Nursing in numbers.

[9] Al Maskari Z, Al Blushi A, Khamis F, Al Tai A, Al Salmi I, Al Harthi H (2021). Characteristics of healthcare workers infected with COVID-19: a cross-sectional observational study. Int J Infect Dis.

[10] Duprat IP, Melo GCD (2020). Análise de casos e óbitos pela COVID-19 em profissionais de enfermagem no Brasil. Rev Bras Saúde Ocupacional.

[11] Sabetian G, Moghadami M, Hashemizadeh FHL, Shahriarirad R, Fallahi MJ, Asmarian N (2021). COVID-19 infection among healthcare workers: a cross-sectional study in southwest Iran. Virol J.

[12] Feingold JH, Hurtado A, Feder A, Peccoralo L, Southwick SM, Ripp J (2021). Posttraumatic growth among health care workers on the frontlines of the COVID-19 pandemic. J Affect Disord.

[13] Von Elm E, Altman DG, Egger M, Pocock SJ, Gotzsche PC, Vandenbroucke JP, STROBE Initiative (2008). The Strengthening the Reporting of Observational Studies in Epidemiology (STROBE) statement: guidelines for reporting observational studies. J Clin Epidemiol.

[14] Wang X, Zhenshun Cheng Z (2020). Cross-sectional studies: strengths, weaknesses, and recommendations. Chest.

[15] Gottlieb M, Sansom S, Frankenberger C, Ward E, Hota B (2020). Clinical course and factors associated with hospitalization and critical illness among COVID-19 patients in Chicago, Illinois. Acad Emerg Med.

[16] Sant&apos;ana G, Imoto AM, Amorim FF, Taminato M, Peccin MS, Santana LA (2020). Infection and death in healthcare workers due to COVID-19: a systematic review. Acta Paul Enferm.

[17] Chou R, Dana T, Buckley DI, Selph S, Fu R, Totten AM (2020). Epidemiology of and risk factors for Coronavirus infection in health care workers: a living rapid review. Ann Intern Med.

[18] Sanchez-Taltavull D, Castelo-Szekely V, Murugan S, Hamley JID, Rollenske T, Ganal-Vonarburg SC (2021). Regular testing of asymptomatic healthcare workers identifies cost-efficient SARS-CoV-2 preventive measures. PLoS One.

[19] World Health Organization (2020). Prevention, identification and management of health worker infection in the context of COVID-19 - Interim guidance..

[20] Shah ASV, Gribben C, Bishop J, Hanlon P, Caldwell D, Wood R (2021). Effect of vaccination on transmission of COVID-19: an observational study in healthcare workers and their households.. medRxiv.

[21] Coccia M (2022). Preparedness of countries to face covid-19 pandemic crisis: Strategic positioning and underlying structural factors to support strategies of prevention of pandemic threats. Environ Res.

[22] Aranaz-Andrés JM, McGee-Laso A, Galán JC, Cantón R, Mira J (2021). Activities and Perceived Risk of Transmission and Spread of SARS-CoV-2 among Specialists and Residents in a Third Level University Hospital in Spain. Int J Environ Res Public Health.

[23] Hu M, Lin H, Wang J, Xu C, Tatem AJ, Meng B (2021). Risk of coronavirus disease 2019 transmission in train passengers: an epidemiological and modeling study. Clin Infect Dis.

[24] Vega EAU, Antoniolli L, Macedo ABT, Pinheiro JMG, Dornelles TM, Souza SBC (2021). Risks of occupational illnesses among health workers providing care to patients with COVID-19: an integrative review.. Rev. Latino-Am. Enfermagem.

[25] Peres-Neto J, Souza MF, Barbosa AMC, Marsico LL, Barbieri W, Palacio DC (2021). Factors Associated with SARS-CoV-2 Infection among Oral Health Team Professionals. Pesqui Bras Odontopediatria Clin Integr.

[26] Secretaria de Estado da Saúde de Alagoas (BR) (2021). Governo autoriza transporte para mais de mil profissionais da área de saúde. https://www.saude.al.gov.br/governo-autoriza-transporte-para-mais-de-mil-profissionais-da-area-de-saude.

[27] Albaqawi HM, Pasay-An E, Mostoles R, Villareal S (2021). Risk assessment and management among frontline nurses in the context of the COVID-19 virus in the northern region of the Kingdom of Saudi Arabia. Appl Nurs Res.

[28] Norbert S, Birkenfeld AL, Schulze MB (2021). Global pandemics interconnected - obesity, impaired metabolic health and COVID-19. Nat Rev Endocrinol.

[29] Augustine RSA, Nayeem A, Salam SA, Augustine P, Dan P, Monteiro P (2022). Increased complications of COVID-19 in people with cardiovascular disease: Role of the renin-angiotensin-aldosterone system (RAAS) dysregulation. Chem Biol Interact.

[30] Musheyev B, Janowicz R, Borg L, Matarlo M, Boyle H, Hou W (2021). Characterizing non-critically ill COVID-19 survivors with and without in-hospital rehabilitation. Sci Rep.

[31] Murugan C, Ramamoorthy S, Kuppuswamy G, Murugan RK, Sivalingam Y, Sundaramurthy A (2021). COVID-19: A review of newly formed viral clades, pathophysiology, therapeutic strategies and current vaccination tasks. Int J Biol Macromol.

[32] Guan W, Ni Z, Hu Y, Liang W, Ou C, He JX (2020). Clinical characteristics of coronavirus disease 2019 in China. N Engl J Med.

[33] Díez-Manglano J, Solís-Marquínez MN, Álvarez García A, Alcalá-Rivera N, Maderuelo Riesco I, Gericó Aseguinolaza M (2021). Healthcare workers hospitalized due to COVID-19 have no higher risk of death than general population. Data from the Spanish SEMI-COVID-19 Registry. PloS One.

[34] Grasselli G, Greco M, Zanella A, Albano G, Antonelli M, Bellani G (2020). Risk factors associated with mortality among patients with COVID-19 in intensive care units in Lombardy, Italy. JAMA Intern Med.

[35] Scott H, Zahra A, Fernandes R, Fries BC, Thode HC, Singer AJ (2022). Bacterial infections and death among patients with Covid-19 versus non Covid-19 patients with pneumonia. J Emerg Med.

[36] Rebmann T, Vassallo A, Holdsworth JE (2021). Availability of personal protective equipment and infection prevention supplies during the first month of the COVID-19 pandemic: A national study by the APIC COVID-19 task force. Am J Infect Control.

